# Description of a concurrent thyroid haemangioma and a follicular adenoma

**DOI:** 10.4102/sajr.v28i1.2863

**Published:** 2024-07-31

**Authors:** Liezel Coetzee, Wilhelmina Conradie, Razaan Davis, Rubina Razack

**Affiliations:** 1 Division of Anatomical Pathology, Department of Pathology, National Health Laboratory Service, Faculty of Medicine and Health Sciences, Tygerberg Hospital and Stellenbosch University, Cape Town, South Africa; 2 Division of General Surgery, Department of Surgery, Faculty of Medicine and Health Sciences, Tygerberg Hospital and Stellenbosch University, Cape Town, South Africa; 3 Division of Radiodiagnosis, Department of Medical Imaging and Clinical Oncology, Faculty of Medicine and Health Sciences, Tygerberg Hospital and Stellenbosch University, Cape Town, South Africa

**Keywords:** vascular proliferations, thyroid, ISSVA classification, radiology, pathology

## Abstract

Thyroid gland vascular proliferations pose a challenge to pathologists, radiologists and surgeons. This is illustrated in the case of a 36-year-old woman who underwent a diagnostic thyroid lobectomy for a suspected follicular neoplasm according to cytology. Histological diagnosis confirmed a follicular adenoma as well as a cavernous haemangioma or venous malformation.

**Contribution:**

Thyroid vascular lesions are rare. Evolving nomenclature and application of the International Society for the Study of Vascular Anomalies classification are discussed. Pertinent radiological clues are highlighted to pre-empt the diagnosis and avoid potential surgical complications.

## Introduction

The spectrum of vascular proliferations in the thyroid includes reactive proliferative changes, benign to malignant tumours and a range of malformations. These lesions are considered controversial in thyroid pathology, as consistency in nomenclature and aetiopathogenesis is lacking, particularly so for benign proliferations.^1,2,3^ Further contention arises when these lesions occur secondary to haemorrhage in a nodular goitre or following a fine-needle aspiration biopsy (FNAB) procedure field.^1,3^

A case of a vascular lesion is presented, initially discovered by histological evaluation of a thyroid lobectomy resection specimen performed for nodular thyroid disease and confirmed by retrospective review of the ultrasound (US) images. Although traditionally regarded as a cavernous haemangioma, this report discusses the evolving nomenclature and aetiopathogenesis in the field of vascular pathology as applied to the thyroid gland. Because of their low incidence and non-specific features on US, thyroid haemangiomas are considered a challenging preoperative diagnosis.^4^ Pertinent radiological clues are highlighted to pre-empt the diagnosis and avoid potential surgical complications.

## Ethical considerations

Ethical clearance to conduct this study was obtained from the Stellenbosch University, Health Research Ethics Committee (No. C22/11/039).

## Patient presentation

**FIGURE 1:**
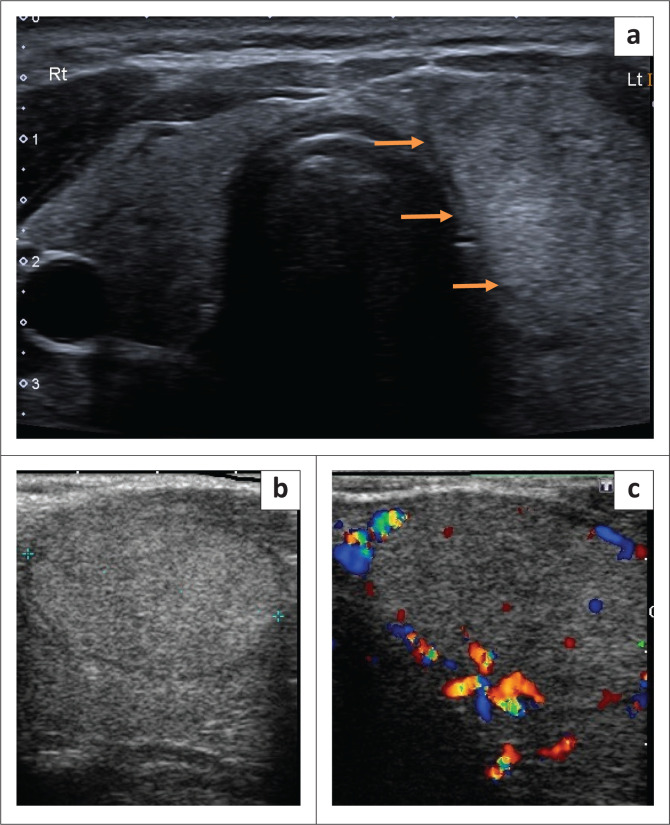
(a) Transverse view ultrasound image of the thyroid gland shows a well-defined, isoechoic, oval nodule with a thin peripheral halo (orange arrows) in the left lobe. (b and c) Longitudinal view ultrasound. The lesion in the left lobe of the thyroid gland (b) demonstrates predominantly peripheral flow on colour flow Doppler (c).

**FIGURE 2:**
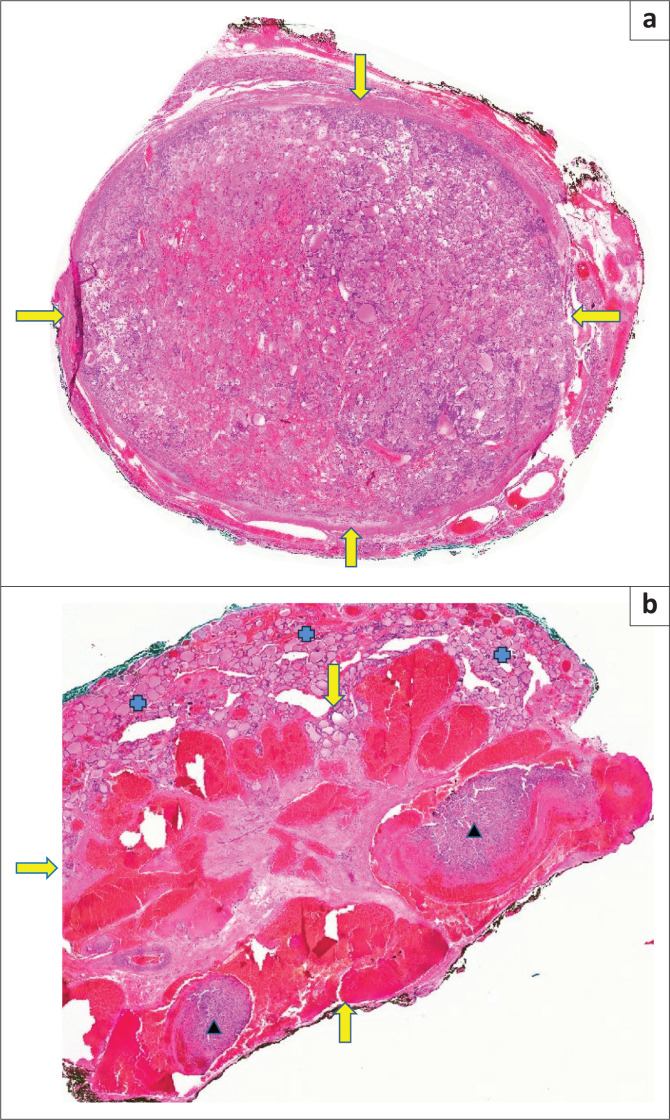
(a) Hematoxylin and eosin (H&E) stained section of the encapsulated follicular adenoma (yellow arrows). (b) H&E section of the vascular lesion (yellow arrows) comprising large cystic, thin-walled, blood-filled spaces, containing organising thrombi (black triangles), separated by connective tissue with background thyroid tissue (blue crosses).

A 36-year-old woman, whose father was known to have thyroid cancer, presented with a 3-year history of a progressively enlarging left neck mass, causing intermittent shortness of breath. Ultrasound findings showed a 43 mm × 41 mm × 27 mm solid, isoechoic homogenous nodule with a thin peripheral halo (Figure 1a) and predominantly peripheral flow (Figure 1b and c), which was aspirated and diagnosed as a Bethesda IV category: consistent with a follicular neoplasm or suspicious for a follicular neoplasm. A diagnostic left thyroid lobectomy was performed and on macroscopic inspection, a dominant nodule measuring 45 mm × 35 mm was found with a tan and haemorrhagic cut appearance. On microscopic evaluation, a follicular adenoma was present with a mixed micro- and macrofollicular growth pattern, measuring 45 mm × 35 mm (Figure 2a).

**FIGURE 3:**
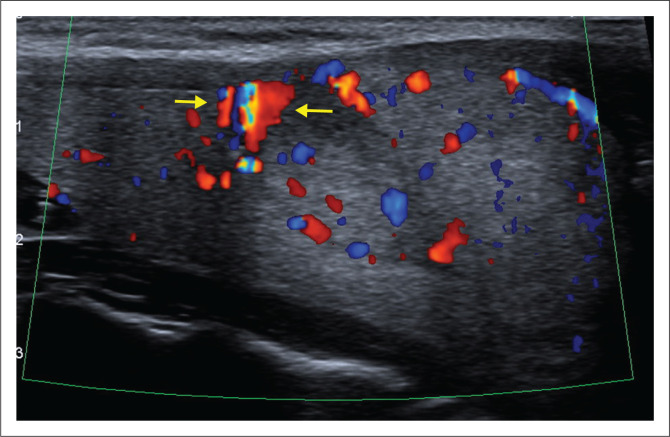
Colour flow Doppler ultrasound (longitudinal view). Focal area of increased flow (yellow arrows) superior and adjacent to the well-defined larger isoechoic nodule.

In addition, a vascular lesion measuring 5 mm × 8 mm was identified immediately superior to the follicular adenoma, comprising large cystic, thin-walled, blood-filled spaces, lined by flattened cells and separated by connective tissue (Figure 2b). Organising thrombi were evident in some of the spaces. Absence of elastic fibers on the Van Gieson’s stain and positive endothelial cells with CD31 immunohistochemistry confirmed these spaces to be venous in nature. The morphological picture was in keeping with what was historically referred to as a cavernous haemangioma. A retrospective review of the US images identified a focal area of increased flow superior and adjacent to the well-defined larger isoechoic nodule (Figure 3), that correlated with the lesion identified on the histological specimen.

### Management and outcome

The patient had an uneventful post-operative course, and was discharged after short term follow-up.

## Discussion

Neoplastic vascular proliferations in the thyroid gland are rare, and malformations even more so, with just under 40 cases published to date.^4,5,6,7,8,9,10,11,12^ From the literature review, all the neoplastic vascular proliferations were called haemangioma, some qualified by a descriptor of cavernous, and fewer as capillary.^4,5,6,7,8,9,10,13,14,15,16^ The term ‘haemangioma’ is non-specific and, in some ways, a misnomer. It refers to a vast array of potential benign vascular anomalies that give the impression by using the suffix ‘oma’ that it is neoplastic in nature when this may not necessarily be the case.

To standardise terminology, the International Society for the Study of Vascular Anomalies (ISSVA) established a basic and systematic classification of vascular anomalies. The ISSVA broadly groups vascular lesions into two domains: vascular tumours with a spectrum from benign to malignant, and vascular malformations classified as non-neoplastic (Table 1). The latter is further divided according to the type of vessels involved (capillary, lymphatic, venous, arteriovenous) and their respective complexities.^13,14,17^ According to this classification, cavernous haemangioma falls under the category venous malformations. With the ISSVA classification, vascular malformations are also divided into haemodynamic flow patterns, that is, no flow to low flow versus high flow. A venous malformation is typically low flow. In this case, Doppler spectral flow was not captured.

**TABLE 1:** Overview table of the International Society for the Study of Vascular Anomalies classification.

Anomaly	Subtype	Discription
Vascular tumours	Benign Locally aggressive or borderline Malignant	
Vascular malformations	Simple (based on the type of blood vessel)	Capillary malformations Lymphatic malformations Venous malformations Arteriovenous malformations† Arteriovenous fistula†
	Combined	Defined as two or more vascular malformations found in one lesion
	Major named vessels	Abnormalities in the origin and/or course and/or number of major blood vessels that have anatomical names
	Associated with other anomalies	Syndromes in which vascular malformations are complicated by symptoms other than vascular anomalies including soft tissue or skeletal abnormalities

*Source*: Adopted from Kunimoto K, Yamamoto Y, Jinnin M. ISSVA classification of vascular anomalies and molecular biology. Int J Mol Sci. 2022;23(4):2358. https://doi.org/10.3390/ijms23042358

† , high flow lesions.

The current World Health Organization (WHO) Classification of Endocrine Tumours still uses the term haemangioma with cavernous haemangioma forming one of many other subtypes.^2^ Although the authors do state that haemangiomas should be regarded as a non-neoplastic vascular malformation, further clarification is not provided.^2^

The thyroid gland, like other endocrine organs, is markedly vascular, making it prone to haemorrhage, haematoma formation with organising fibrosis and endothelial reparative injury. Excessive bleeding can give rise to secondary haemangiomas in that vascular proliferation (neovascularisation) takes place attempting to organise the haematoma, also described as pseudomalformations.^6^ Other differential diagnoses that should be considered include granulation tissue, reactive or malformative vascular polyps and papillary endothelial hyperplasia.^2^ These regressive changes make diagnosing a vascular lesion in the thyroid gland contentious. We believe that the haemangioma described in the presented case was a primary lesion. Justification for this is that it was distinct from the follicular adenoma, was not associated with any reparative type of injury and was evident on US before the FNAB.

Preoperative diagnosis of vascular lesions in the thyroid is challenging as there are no distinctive signs on ultrasonography.^4^ A lower diagnostic awareness exists as these lesions are infrequently encountered. Some authors have suggested that thyroid haemangiomas can be correctly diagnosed preoperatively as well-circumscribed, compressible hypoechoic lesions with multiple, internal linear septations because of the presence of multiple vascular channels.^4,8^ Slow flow and dilated vascular channels predispose to thrombosis and phlebolith formation, noticed by coarse calcification.^8^ These features were not identified on the greyscale US images in our case, most likely because the lesion was small. As with most cited reports, a confident diagnosis of a haemangioma or venous malformation could only be made on post-surgical pathological examination.^4,5,6,7,8,9,10,11,12^

We conclude by emphasising using current terminology as recommended by the ISSVA to ensure reproducibility from a pathological nomenclature perspective and to correlate with appropriate radiological haemodynamic flow studies. Although it may seem inconsequential in this case, accurate terminology is important in other settings as the natural history and treatment options vary depending on the type of vascular anomaly.^15,16^ In some cases, natural involution with minimal intervention is needed, compared to other lesions where possible preoperative therapeutic interventions like sclerotherapy and oral propanol can reduce surgical risk and postoperative complications. We agree with others that the term ‘haemangioma’ is too often used and incorrectly so, as is evident in the literature review titles.^4,5,6,7,8,9,10,15,16^

## Conclusion

This case illustrates a rare entity of a primary thyroid cavernous haemangioma or venous malformation incidentally and concurrently diagnosed with a dominant follicular adenoma. The controversy surrounding vascular proliferations of the thyroid gland can be resolved with increased awareness of diagnostic pitfalls in pathology and appropriate clinical pathological correlation with imaging in a multidisciplinary team. The application of the ISSVA classification for all vascular lesions is encouraged for future case reports and research to improve reproducibility in the nomenclature of vascular lesions, including that of the thyroid.
